# Cell-free DNA from plasma as a promising alternative for detection of gene mutations in patients with Maffucci syndrome

**DOI:** 10.1186/s41065-022-00223-2

**Published:** 2022-01-18

**Authors:** Yi Sun, Xindong Fan, Yamin Rao, Zhenfeng Wang, Deming Wang, Xitao Yang, Lianzhou Zheng, Mingzhe Wen, Ren Cai, Lixin Su

**Affiliations:** 1 Department of Interventional Therapy, Multidisciplinary Team of Vascular Anomalies, Shanghai Ninth People’s hospital, Shanghai Jiao Tong University, Shanghai, People’s Republic of China; 2 Department of pathology, Shanghai Ninth People’s hospital, Shanghai Jiao Tong University, Shanghai, People’s Republic of China

**Keywords:** Maffucci syndrome, Cell-free DNA, IDH1 mutation, Spindle cell hemangioma, Enchondroma

## Abstract

Maffucci syndrome (MS, OMIM 166000) is an extremely unusual, nonhereditary, multisystemic disorder that is characterized with multiple enchondromas and vascular lesions, most of which are spindle cell hemangiomas. Complications of MS, such as bone deformities and dysfunction caused by enchondromas, usually increase during childhood and adolescence. Malignant transformation of enchondromas and other malignancies are the most severe complications. MS is caused by somatic mosaic IDH1/2 mutations, 65% of which are the IDH1 p.Arg132Cys variant. Due to its rarity, there is no international consensus for the most appropriate treatment option of MS.

Here, we report a case of a female patient presenting with multiple enchondromas and spindle cell hemangiomas (SCHs) on bilateral hand and feet diagnosed as MS. A detailed clinical, pathological and genetic diagnosis of MS was rendered. Integrative Genomics Viewer (IGV) visualization of next-generation sequencing (NGS) data revealed the consistent detection of the low-frequency somatic IDH1 p.Arg132Cys mutation between SCH tissue and cystic blood-derived cfDNA. This is the first successful molecular diagnosis of MS complicated with SCH utilizing minimally invasive cfDNA techniques. We suggest that cfDNA sequencing could potentially be used as an alternative, reliable and sensitive method to identify molecular information for genetic diagnosis and for future targeted therapies of MS.

## Background

Maffucci syndrome (MS, OMIM 166000) is an extremely unusual, nonhereditary, multisystemic disorder that is characterized with multiple enchondromas and vascular lesions, most of which are spindle cell hemangiomas [1]. Enchondromas are benign tumors caused by chondrocytes proliferations involving the long bones and are mainly bilaterally distributed in limbs [2]. Spindle cell hemangiomas (SCHs) are nonneoplastic reactive vascular lesions involving mainly soft tissues, and they may cause pain and dysfunction because of rapid progression and thrombosis [3]. In addition to the characteristic multiple enchondromas and vascular malformations, patients with MS also exhibit related bone asymmetry, deformities and functional defects of the affected limbs, such as bony distortion, scoliosis, short stature and pathologic fracture [4, 5]. Moreover, according to the existing literature, the risk of many malignancies, including chondrosarcomas, gliomas, ovarian tumors and other sarcomas is significantly increased in patients with MS [6–9]. Due to its rarity, there is no international consensus for the most appropriate treatment option of MS. Conventional management includes conservative management, surgical excision and long-term screening for malignant transformations [10].

MS is caused by somatic mosaic IDH1/2 mutations, 65% of which are the IDH1 p.Arg132Cys variant [11, 12]. Consequently, molecular diagnosis requires surgically excised enchondroma or SCH tissue. More sensitive and noninvasive diagnostic methods may provide adequate information for molecular characterization. In recent years, the clinical utility of cell-free DNA (cfDNA) has been explored in many kinds of vascular malformations [13–16]. However, there is currently no report of cfDNA genetic analysis on MS associated with SCH.

Here, we report a successful molecular diagnosis of MS complicated with SCH utilizing minimally invasive cfDNA techniques. Integrative Genomics Viewer (IGV) visualization of next-generation sequencing (NGS) data revealed the consistent detection of the low-frequency somatic IDH1 p.Arg132Cys mutation between SCH tissue and cystic blood-derived cfDNA, supporting the hypothesis that cell-free DNA from plasma could be a promising alternative for tissue sample to promote molecular diagnosis of MS.

## Case report

**Fig. 1:**
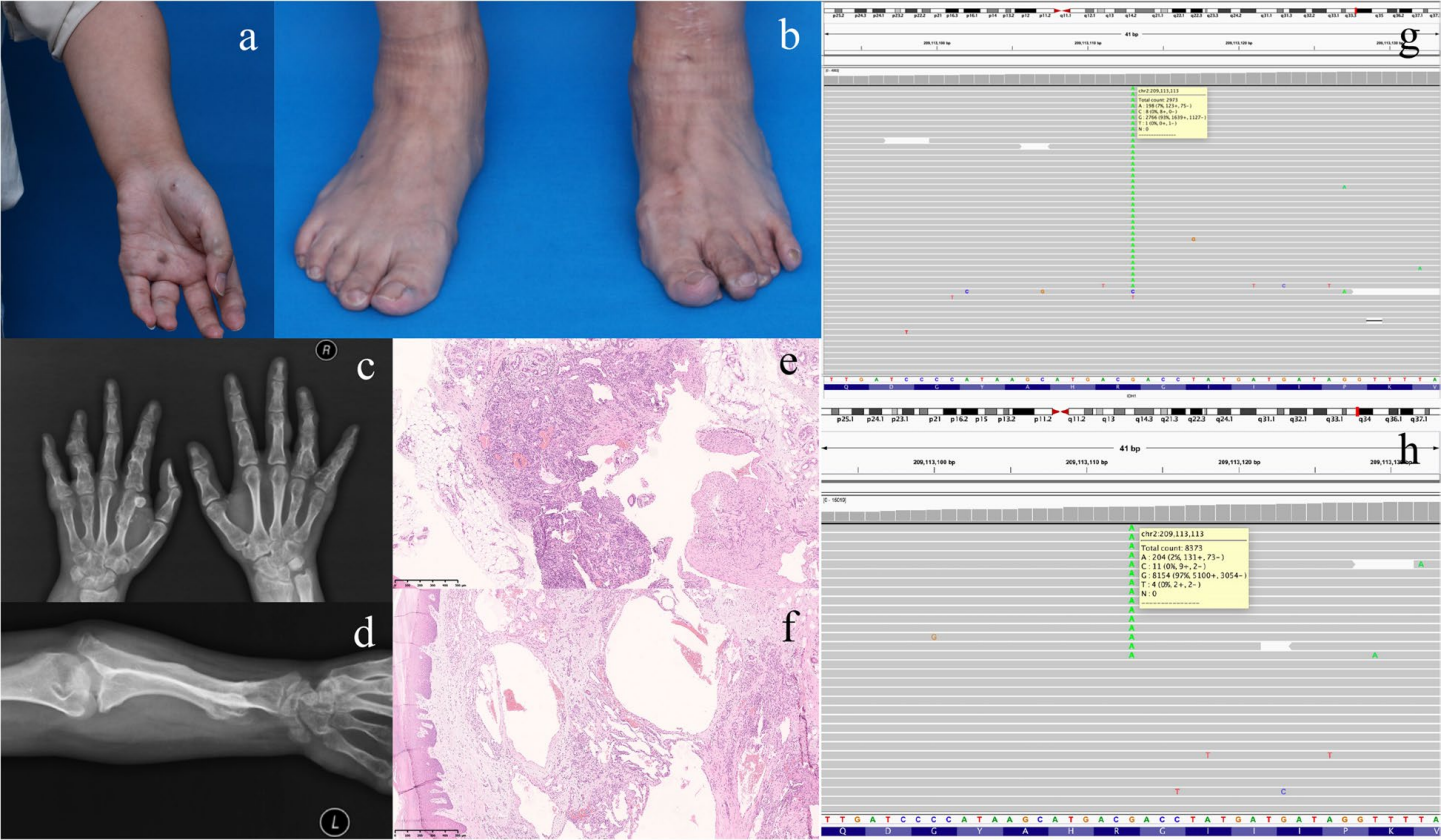
**a-b:** Clinical manifestation of the patient. Multiple exophytic, round, compressible bluish-purple nodules. Figure 1b, arrow: subtotal resection site of the feet SCH lesions. **c-d**: Radiography revealed multiple enchondromas at bilateral phalanges (Fig. 1c) and the left distal ulna (Fig. 1d). **e-f**: Histological analysis showed that the tumor consisted of bland spindle cell proliferations (Fig. 1e) and dilated, slit-like, thin-walled veins involving the superficial and deep layers of the dermis (Fig. 1f), confirming the diagnosis of SCH (hematoxylin–eosin). **g-h**: IGV visualization of NGS data for the SCH tissue (Fig. 1g) and cystic blood (Fig. 1h) with somatic mutation in IDH1 p.Arg132Cys

A 24-year-old female came to our hospital with multiple, round, bluish-purple masses on bilateral hand and feet. The patient disclosed a medical history in which the masses gradually increased and enlarged. However, the lesions recurred rapidly and progressed with pain and walking dysfunction. The patient had undergone several surgical excisions to relieve pain. Physical examination showed multiple exophytic, round, compressible bluish-purple nodules (Fig. 1a,b). Radiography revealed multiple enchondromas at bilateral phalanges and the left distal ulna (Fig. 1c,d). Subtotal resection of the feet SCH lesions (Fig. 1b, arrow) was performed to relieve the pain, and after which, the patient’s symptoms gradually improved.

In order to further clarify the diagnosis of SCH and identify genetic changes, we collected tissue samples as well as blood samples from the SCH cysts during the resection to promote targeted next-generation sequencing (NGS). We designed the targeted gene panel based on the classification of vascular anomalies proposed by ISSVA [17].

Histopathological analysis of the excised mass showed that the tumor consisted of bland spindle cell proliferations and dilated, slit-like, thin-walled veins involving the superficial and deep layers of the dermis, confirming the diagnosis of SCH (Fig. 1e,f).

The molecular analysis showed that both the SCH tissue and cystic blood had somatic IDH1 p.Arg132Cys mutations. The NGS revealed that IDH1 mutations were identified with allele loads of 6.46 and 2.41% in the tissue and cystic blood samples, respectively. IGV visualization of NGS data for the SCH tissue and cystic blood with somatic mutation in IDH1 p.Arg132Cys is present in Fig. 1g,h. Since the clinical manifestations of multiple enchondromas and SCHs raises the suspicion of Maffucci syndrome and the genetic analysis revealed somatic IDH1 p.Arg132Cys mutations in the SCH tissue and cystic blood samples, we diagnosed the patient with Maffucci syndrome.

## Discussion

Maffucci syndrome (MS), according to the ISSVA classification, is a rare multisystemic malformation of mesodermal tissue, mainly vascular and bony lesions [1, 4]. MS is characterized by multiple enchondromas complicated with vascular malformations (mainly spindle cell hemangiomas (SCHs)) [3]. Enchondromas are common benign bone tumors of the distal extremities. Enchondromas are characterized by chondrocytes proliferations involving the long bones throughout development [2]. Enchondromas usually present as sporadic isolated lesions, but it can also appears as multiple lesions in MS and Ollier’s disease. Enchondromas have a tendency of recurrence, and cause destruction of local bones, leading to pain, bone fractures and other complications. Very few lesions can malignant into chondrosarcomas. SCHs are relatively rare benign vascular tumors often occurred sporadically or in combination with other vascular malformations, such as Klippel–Trenaunay syndrome and MS. SCHs often present on the skin of distal extremities, and can also involve mucosal tissue, such as the oral cavity. SCH usually present and develop in children and adolescents, and have a tendency to recur locally. Due to the dilated veins, formation of thrombus or phlebolith, SCH can result in progressive pain and limb dysfunction. Complications of MS, such as bone deformities and dysfunction caused by enchondromas and SCHs, usually increase during childhood and adolescence. Malignant transformation of enchondromas and other malignancies are the most severe complications.

Pansuriya and Amary et al. reported the identification of somatic mosaic gain-of-function IDH1/IDH2 mutations in MS patients at the same time in 2011, and these mutations have also been also related to isolated enchondromas and chondrosarcomas [11, 12]. Subsequently, the same variant was identified as a hot spot mutation in patients with sporadic and multiple SCHs without enchondromas. Based on the high specificity of IDH gene mutations in SCHs, the identification of these mutations, most of which are the IDH1 p.Arg132Cys variant, can facilitate diagnosis and differential diagnosis from other vascular tumors such as Karposi’s sarcoma. However, there are several limitations in traditional molecular profiling of vascular malformations with tissue samples. First, the reduced sensitivity and bias of mutation detection caused by tissue heterogeneity associated with tissue biopsy is the main disadvantage that cannot be overlooked [18]. In addition, tissue biopsies may be unsafe, infeasible, or otherwise unsuccessful, and they may result in serious side-effects and complications, such as critical bleeding. In this case, a new method with minimal invasion and superior sensitivity was established for the genomic profiling of vascular malformations. Cell-free DNA (cfDNA), also named as liquid biopsy, may enable to provide an accurate diagnostic approach in various clinical fields that avoid the intractable complications associated with tissue biopsy.

In 1948, cell-free DNA (cfDNA) was firstly reported by Mandel and Metais [19]. cfDNA in the serum and plasma has been revealed to be higher in cancer patients than in healthy individuals [20, 21] and has been an active research area in many disciplines of medicine. As a potential biomarker, cfDNA is readily accessible, repeatable, and reliable. Ozeki et al. identified NRAS oncogenic variants not only in tissue samples but also in plasma and pleural effusion, which is the first study of cfDNA in vascular anomalies [22]. Recently, this observation has introduced the possibility of noninvasive diagnostic evaluation for vascular anomalies. K Zenner extracted cfDNA from the plasma and cystic fluid of patients with venous, lymphatic, and arteriovenous malformations, and variants were detected for the first time [13]. Subsequent studies of the application of cfDNA in arteriovenous malformations and Klippel–Trenaunay syndrome further validated the investigation of cfDNA-based molecular diagnostics and provided a noninvasive method to initiate targeted therapy for patients with vascular malformations [14, 15]. However, there is currently no report of cfDNA genetic analysis on MS associated with SCH.

In this study, we report a female patient presenting with multiple enchondromas and SCHs on bilateral hand and feet diagnosed as MS. IDH1 p.Arg132Cys variants were detected in both the SCH tissue and cystic blood-derived cfDNA for the first time. We hypothesized that the proliferated spindled cells composed of endothelial cells, pericytes and fibroblasts drove cfDNA entering the circulation. Moreover, the contact between the proliferated endothelial cells and the systemic circulation in the SCH lesion is higher than that in the peripheral, which also explains the detective sensitivity of the lesion plasma in this study. To our knowledge, this is the first successful molecular diagnosis of MS utilizing minimally invasive cfDNA techniques, suggesting the potential use of a cfDNA-based, noninvasive molecular diagnosis of MS.

The clinical utility of specific IDH1/2 inhibitors has been reported in several malignant cancers. Ivosidenib and enasidenib are targeted inhibitors of the IDH1 and IDH2 proteins, respectively, which have been confirmed to be efficient and tolerable in acute myeloid leukemia (AMLs) in previous studies [23, 24]. Due to the rarity and heterogeneity of clinical phenotypes in MS, the clinical efficacy and risk of IHD1/2 inhibitors in MS has not been explored. Nevertheless, the discovery of IDH1/2 inhibitors encourages further research in MS, especially those with severe manifestations, such as rapid progression of tumors, dysfunction, and malignancy. We assume that these inhibitors may achieve a reduction in tumor growth and reduce the potential malignant risk. Our study provides a noninvasive method to identify molecular information for future targeted therapies.

## Conclusion

We report a case of a female patient presenting with multiple enchondromas and spindle cell hemangiomas (SCHs) on bilateral hand and feet diagnosed as MS. A detailed clinical, pathological and genetic diagnosis of MS was rendered. IGV visualization of NGS data revealed the consistent detection of the low-frequency somatic IDH1 p.Arg132Cys mutation between SCH tissue and cystic blood-derived cfDNA. To our knowledge, this is the first successful genetic diagnosis of MS complicated with SCH utilizing minimally invasive cfDNA techniques. We suggest that cfDNA sequencing could potentially be used as an alternative, reliable and sensitive method for the genetic diagnosis of MS.

## Data Availability

All supporting data of this article are included in the submitted manuscript.

## Abbreviations

MS: Maffucci syndrome
SCHs: Spindle cell hemangiomas
cfDNA: cell-free DNA
IGV: Integrative Genomics Viewer
NGS: Next-generation sequencing
AML: Acute myeloid leukemia
